# Capping Human Water Footprints in the World's River Basins

**DOI:** 10.1029/2019EF001363

**Published:** 2020-02-17

**Authors:** Rick J. Hogeboom, Davey de Bruin, Joep F. Schyns, Maarten S. Krol, Arjen Y. Hoekstra

**Affiliations:** ^1^ Twente Water Centre University of Twente Enschede Netherlands; ^2^ Water Footprint Network Enschede Netherlands; ^3^ Institute of Water Policy, Lee Kuan Yew School of Public Policy National University of Singapore Singapore; ^4^ Deceased November 18, 2019

**Keywords:** water footprint caps, water availability, planetary boundaries, environmental flow requirements, water scarcity, Global Hydrology Models

## Abstract

Increased water demand and overexploitation of limited freshwater resources lead to water scarcity, economic downturn, and conflicts over water in many places around the world. A sensible policy measure to bridle humanity's water footprint, then, is to set local and time‐specific water footprint caps, to ensure that water appropriation for human uses remains within ecological boundaries. This study estimates—for all river basins in the world—monthly blue water flows that can be allocated to human uses, while explicitly earmarking water for nature. Addressing some implications of temporal variability, we quantify trade‐offs between potentially violating environmental flow requirements versus underutilizing available flow—a trade‐off that is particularly pronounced in basins with a high seasonal and interannual variability. We discuss several limitations and challenges that need to be overcome if setting water footprint caps is to become a practically applicable policy instrument, including the need (for policy makers) to reach agreement on which specific capping procedure to follow. We conclude by relating local and time‐specific water footprint caps to the planetary boundary for freshwater use.

## Key Points

- Overexploitation of freshwater resources shows humanity's current inability to live within the means of the planet in terms of water use
- We propose setting water footprint caps per river basin to ensure human water appropriation remains within ecological boundaries
- We relate local and time‐specific water footprint caps to the planetary boundary for freshwater use

## 1 Introduction

Human demand for freshwater for food, feed, fuels, and fibers is putting pressure on water resources in many places around the world. Today, half a billion people live in regions that are affected by year‐round water scarcity (Mekonnen & Hoekstra, 2016). A lack of clean and sufficient freshwater is stalling economic momentum and growth (World Bank, 2016), while overexploitation of water systems undermines biodiversity and resilience of aquatic ecosystems that provide life‐supporting functions (Vörösmarty et al., 2010). Moreover, scarcity of freshwater plays a prominent role in various conflicts and places with social unrest (Gleick, 2019; Kundzewicz & Kowalczak, 2009).

Future projections foresee substantial growth in global water demand (Greve et al., 2018; Kummu et al., 2016). By 2050, nearly half the world's population is estimated to live in places with insufficient land and water resources to meet local demand for food production (Foley et al., 2011; Ibarrola‐Rivas et al., 2017). Concerns have been raised as to whether enough freshwater is available to complete the energy transition under the pathways currently pursued by the International Energy Agency, particularly water availability limiting bioenergy production (Holmatov et al., 2019; Mekonnen et al., 2016). Moreover, failure to restrain humanity's growing water footprint (WF) makes reaching the UN's Sustainable Development Goals (SDGs) a daunting task, not only dedicated SDG6 “to ensure availability and sustainable management of water and sanitation for all” but also other SDGs for which water is foundational (Colglazier, 2015; Jägermeyr et al., 2017).

The key issue concerning humanity's current WF is that it exceeds ecological thresholds in many places, indicating we are not living within our (local) means in terms of water consumption (Hall et al., 2014; Mekonnen & Hoekstra, 2016). An appealing and apparent policy measure to prevent overshoot of limited natural endowments and to reconcile human freshwater appropriation with conservation is to set a blue WF cap at the river basin scale (Hoekstra, 2013; Hoekstra & Wiedmann, 2014). A blue WF cap represents an upper ceiling to total water consumption from renewable groundwater and surface water (i.e., blue water), accounting for the fact that the natural replenishment rate is limited and that part of the water flows need to be reserved for nature. While related notions can be found in literature on environmental flows (Pang et al., 2018; Pastor et al., 2014; Richter, 2010), the explicit idea of setting a WF cap is novel and still in its infancy stage, with only one study exploring its merits (Zhuo et al., 2019). The pioneering work of Zhuo et al. (2019), however, concerned only one basin in China and left many questions unanswered, e.g., regarding the role of temporal variability in availability and uncertainties in estimating runoff and environmental flow requirements (EFR). Initial attempts to formalize a WF cap in policy were made in Australia's Murray‐Darling basin, showing preliminary success as well as several difficulties—again pertaining to issues surrounding temporal variability in water availability (Grafton et al., 2014).

This study aims to propel the discourse on curbing freshwater consumption, by quantitatively investigating potential merits and drawbacks that come with setting WF caps at the basin level. It is the first ever study to estimate monthly blue WF caps for the world's river basins, while addressing implications of temporal variability in availability that emerge once a WF cap is set.

First, maximum sustainable levels of monthly blue water availability (BWA) are estimated by subtracting EFR from natural runoff, for each basin in the world and each month in the period 1970–2005. The term “sustainable” is thus taken to mean “within ecological boundaries” (cf. Costanza et al., 1997). Monthly blue water runoff (BWR) was taken from three state‐of‐the‐art global hydrology models (GHMs) (Wada et al., 2016), of which we calculated an ensemble mean to level out model variability (Haddeland et al., 2011). Likewise, three well‐known methods for establishing EFR (Pastor et al., 2014; Richter et al., 2012; Smakhtin et al., 2004) and their ensemble mean provided us with monthly EFR to be set aside in each basin to guarantee proper aquatic ecosystem functioning (Oki & Kanae, 2006; Vörösmarty et al., 2010). The high spatiotemporal resolution of the models captures interannual and intraannual variability in the resulting water availability levels, allowing the exploration of dynamics that setting a WF cap at a certain historic or average level will likely bring about. Second, therefore, three procedures for formulating a WF cap that are set at varying levels of BWA are presented. Lastly, we address implications of temporal variability in terms of unutilized WF potential and implicitly allowed violations to EFR.

This study presents a major advancement to the field of Water Footprint Assessment (Hoekstra, 2017), by providing a first exploration of how we could formulate WF caps. The study is relevant for developing well‐informed policy at the basin level, by proposing a means to transition away from persistent overshoot toward sustainable consumption of a basin's limited freshwater resources. Moreover, we add to the contemporary discourse on a planetary boundary (PB) for freshwater consumption (Gerten et al., 2013; Rockström et al., 2009; Steffen et al., 2015) by postulating regionalized and time‐specific upper ceilings that must underlie any global annual PB for freshwater consumption (Heistermann, 2017).

## 2 BWA Varies in Space and Time

**Figure 1:**
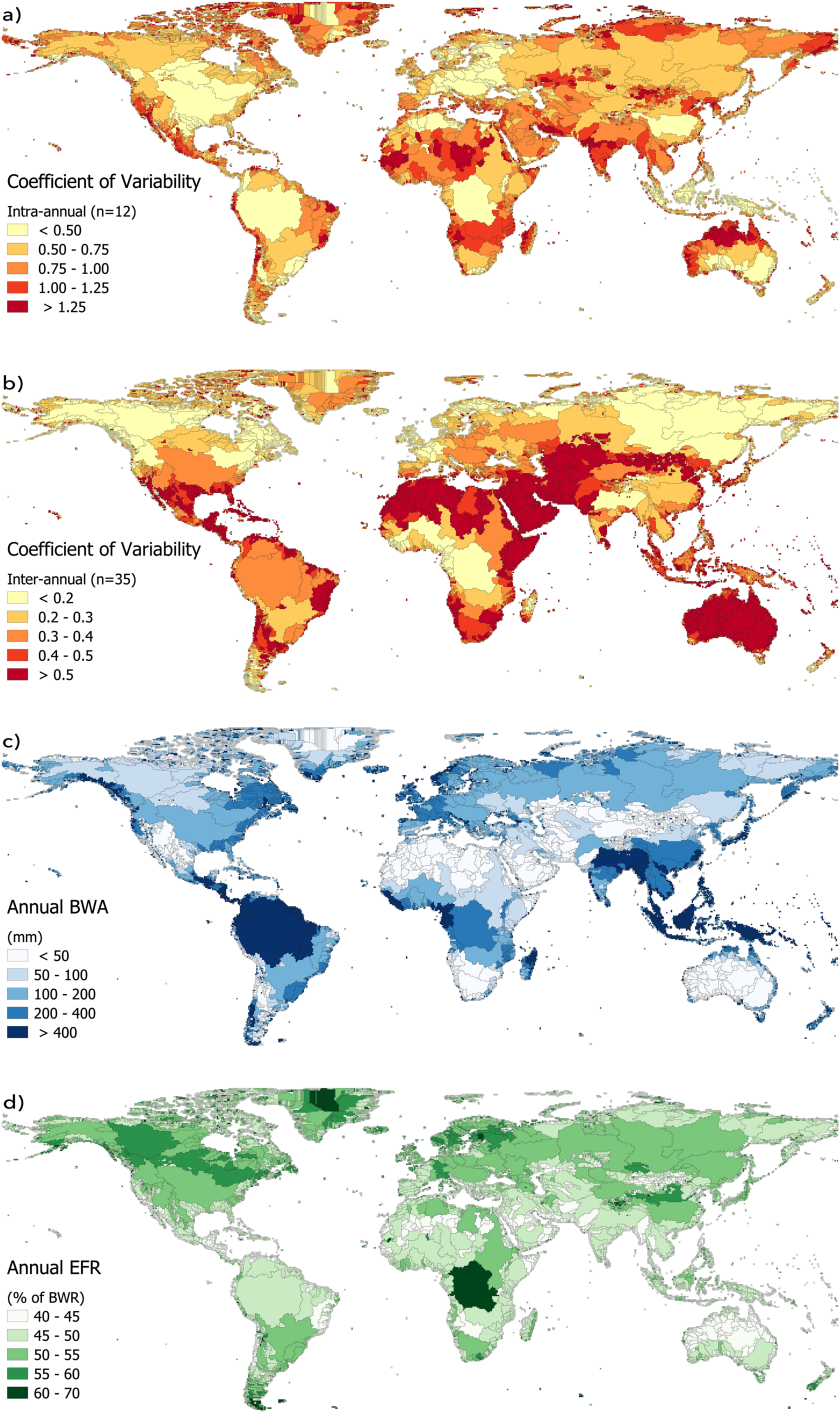
Coefficient of (a) intraannual variation in long‐term average monthly blue water availability (BWA) over the period 1970–2005 (*n* = 12) and (b) interannual variation in annual BWA over the period 1970–2005 (*n* = 35). Darker red basins experience more pronounced variability in BWA within or over the years. The data refer to ensemble means of three global hydrology models and three methods to estimate environmental flow requirements for over 11,000 basins (see section 8). (c) Annual BWA in millimeters for all basins considered, with a global average BWA of 187 mm year^−1^, and (d) EFR as the percentage of BWR on an annual basis, based on ensemble means of both EFR methods and BWR models, with a global average of 49%.

We estimated monthly BWA (m^3^ s^−1^) by subtracting monthly EFR (m^3^ s^−1^) from monthly BWR (m^3^ s^−1^) for each month in the period 1970–2005, for all basins in the world. We found that BWA is unevenly distributed over time, with some basins displaying a relatively constant level of availability, while others exhibit more fickle behavior within and between years (Figures 1a and 1b, respectively). This heterogeneity also arised in space, where both BWA (Figure 1c) and the percentage of BWR that needs to be reserverd for EFR (Figure 1d) vary substantially across basins.

**Figure 2:**
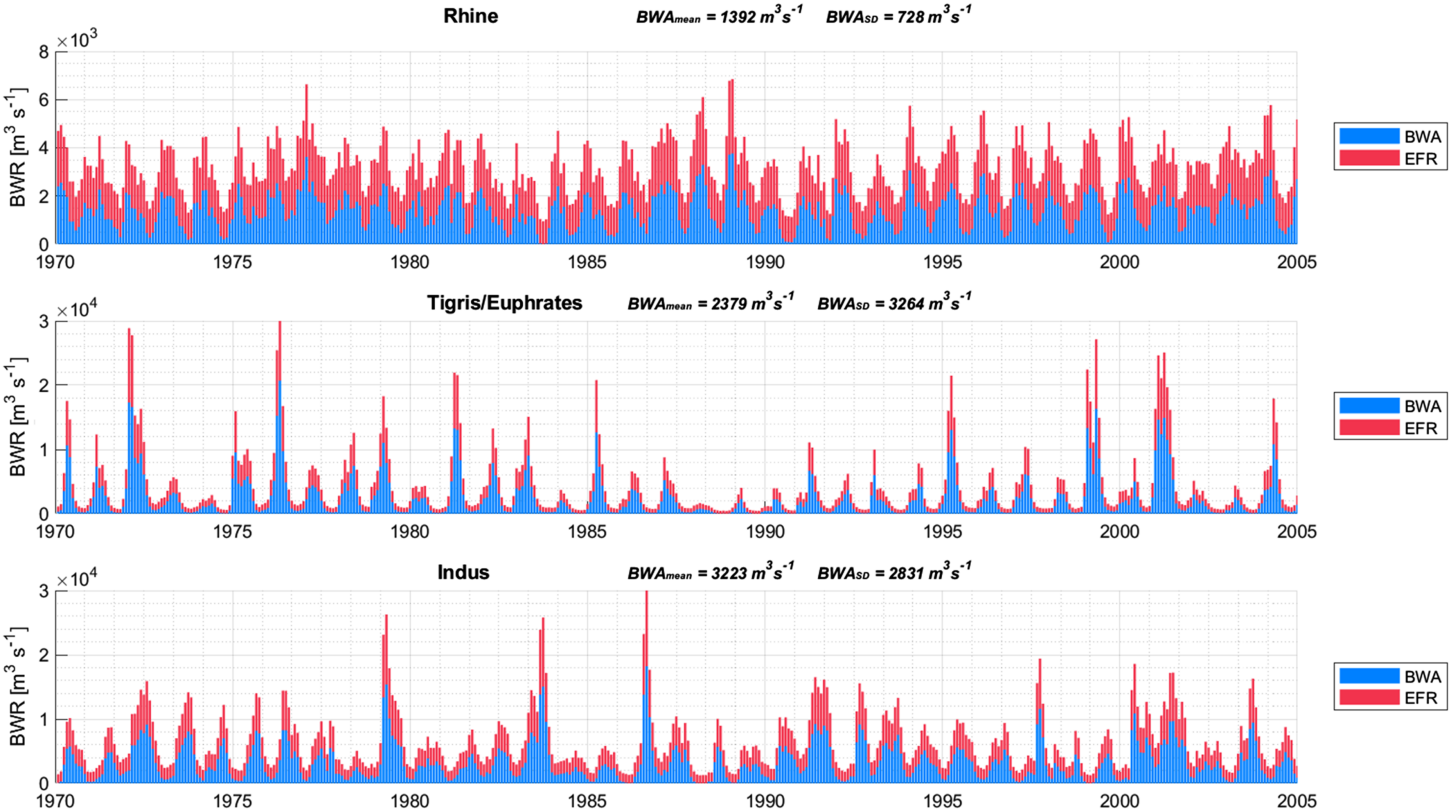
Monthly blue water runoff (BWR) partitioned into environmental flow requirements (EFR) and blue water availability (BWA), for three selected basins, with mean and standard deviation (SD) values of BWA. The data refer to ensemble the means of three global hydrology models and three EFR methods (see section 8).

**Figure 3:**
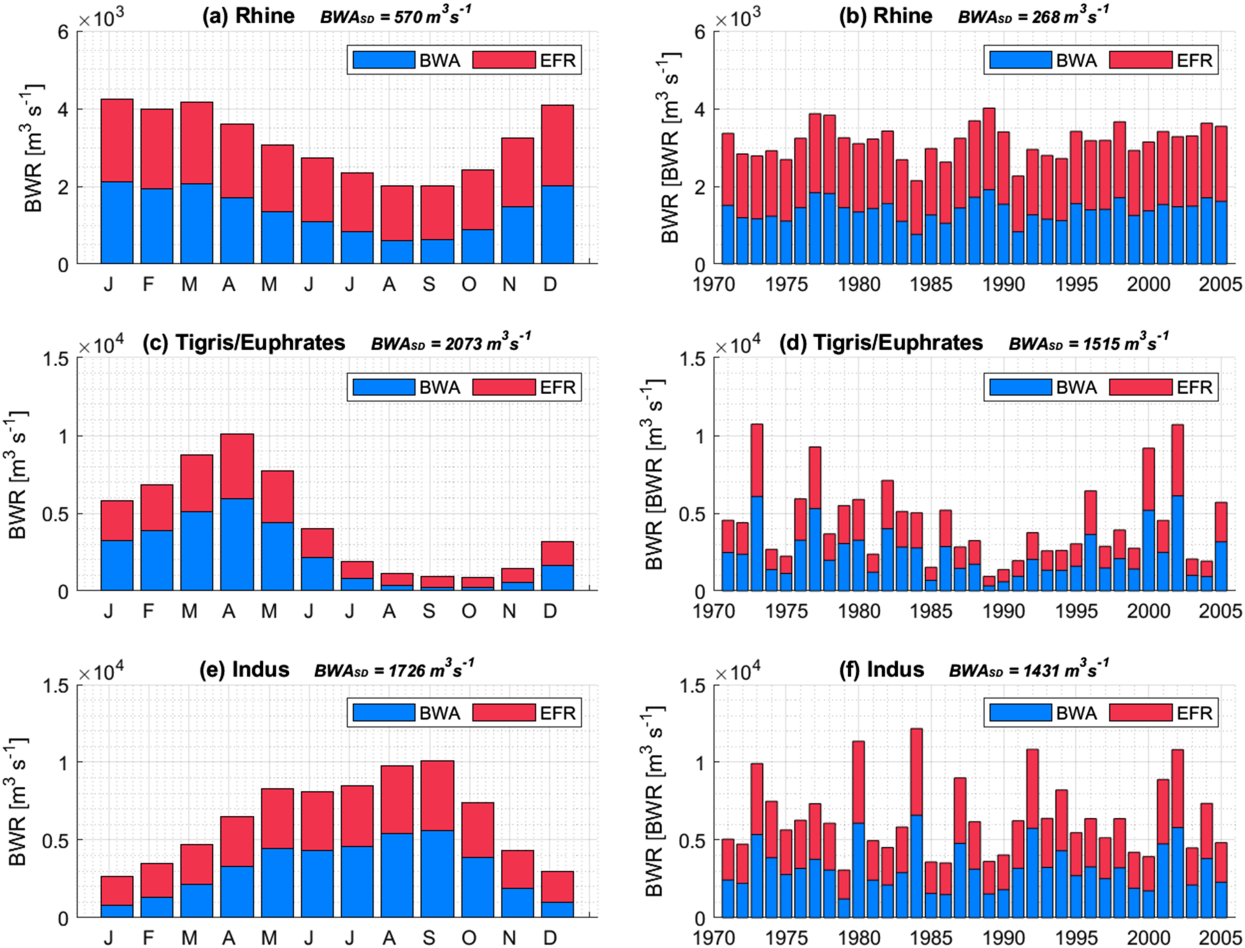
Monthly blue water runoff (BWR) partitioned into environmental flow requirements (EFR) and blue water availability (BWA), for three selected basins, with mean and standard deviation (SD) values of BWA (a, c, e) within an average year and (b, d, f) between years in the period 1970–2005.

Figures 2 and 3 zoom in from the global picture to three selected basins with varying hydrological regimes and characteristics—the rain and snowmelt‐fed Rhine basin, the predominantly semi‐arid Tigris/Euphrates basin with a pronounced wet and dry period, and the monsoonal Indus basin. Where the Rhine shows a relatively constant BWA both throughout the year and over the years, the Tigris/Euphrates and Indus basins display a much larger intraannual and interannual variability. Particularly pronounced periods of extreme low flows, e.g., in the Tigris/Euphrates in the late 1980s, leave little room for human appropriation, and either water shortages or overexploitation seems inevitable (Kavvas et al., 2011). The Rhine basin—and to a lesser extent even the Indus basin—experienced fewer extreme low flow months that can foil continuous allocation of water for human purposes under a capping policy arrangement. Already stressed basins will likely face bigger challenges in reducing WFs to cap values, especially in conjunction with high temporal variability in BWA. Monthly and annual average values of BWA, EFR, and BWR for all basins considered in this study, as well as their coefficients of variation, are provided as a separate data set (Hogeboom et al., 2019).

## 3 Variability Has Implications for Setting WF Caps

The premise of setting a monthly blue WF cap in a river basin is that the sum of consumption over all water using activities in that basin should not exceed the cap. Ideally, threshold values are to be formalized dynamically, so that they will be stricter in months that are relatively dry compared to the long‐term average and less strict in relatively wet months, but herein lies the difficulty that long‐term predictions of runoff (i.e., a seasonal lead, at least) are difficult and surrounded by significant uncertainties. Therefore, a WF cap will have to be based on some historic or average measure of BWA in the basin. Here we distinguish three WF cap options, each one following another procedure. In the three options, caps are set at (a) the long‐term average of the monthly average BWA over the period 1970–2005, (b) the 25th percentile of monthly average BWA, or (c) the minimum monthly BWA that occurred in the period 1970–2005. For each of these cap settings, we addressed—per river basin and given the actual regime of fluctuating water availability—the implications of sticking to the caps on the violation of EFR (in case of relatively dry periods) or on the underutilization of WF potential (in case of relatively wet periods).

**Figure 4:**
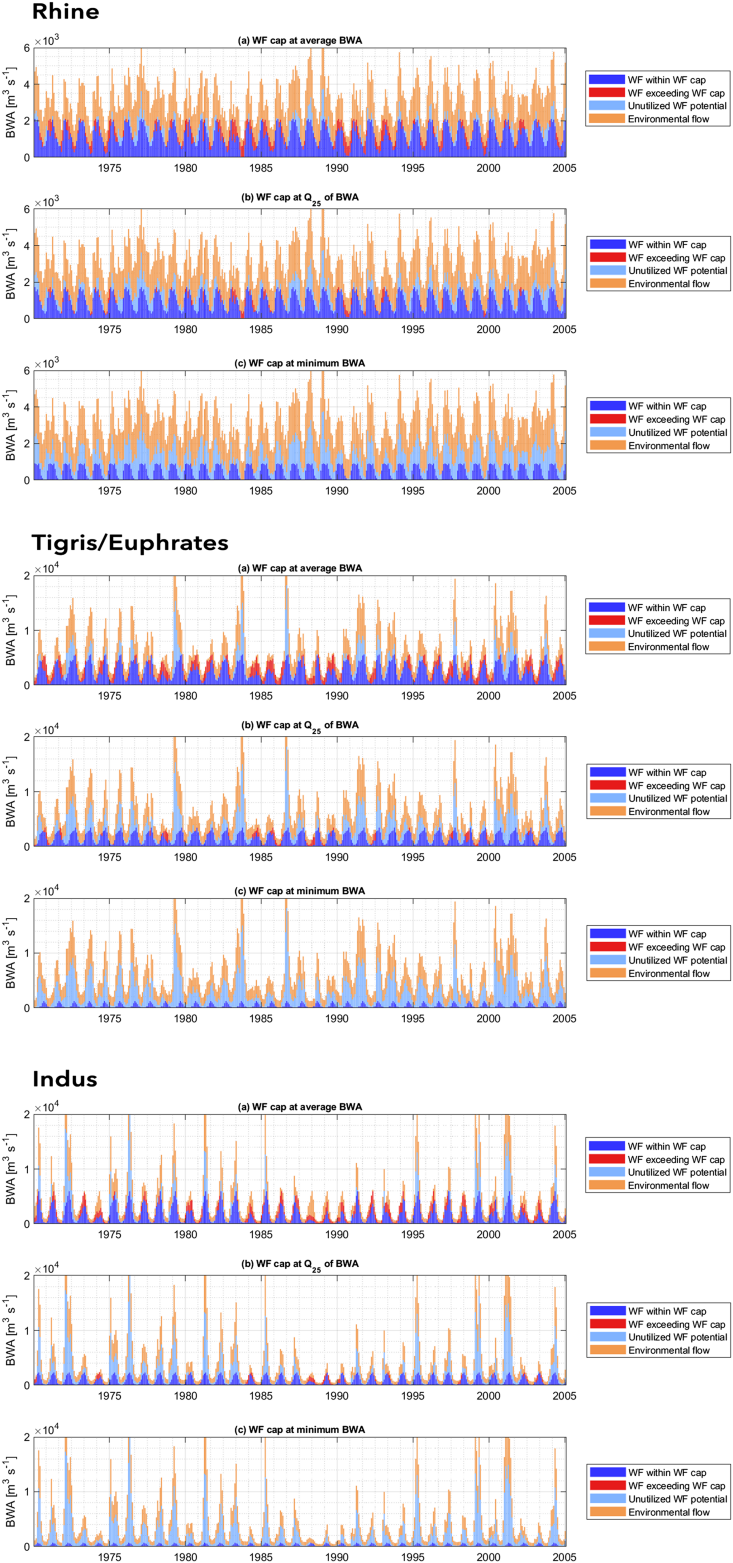
The implications of three WF cap options for the (top) Rhine, (middle) Tigris/Euphrates, and (bottom) Indus basins, where WF caps are set at average (a) BWA, (b) Q25 of BWA, or (c) minimum BWA, assuming actual WFs in the basins are equal to the WF cap.

Figure 4 illustrates the implications of setting a monthly WF cap according to the three procedures for the Rhine, Tigris/Euphrates, and Indus basins. In order to test the consequence of using WF caps, it is assumed that in every river basin WFs are allocated and realised up to the governing cap. For all three WF cap options, it shows that if in a given month BWA is higher than the WF cap set for that month, environmental flows will not be violated (WFs in dark blue are fully within BWA). At the same time, these months leave an unutilized WF potential, since any BWA beyond the cap is not allocated to human consumption (light blue). In wet months in which BWA exceeds the cap, an unutilized WF potential is inevitable, but underutilizing available flow in dry months in which BWA still exceeds the cap can be undesirable. If BWA is lower than the WF cap set for a particular month, utilizing BWA to its full extent means that EFR will be partly compromised (red stacks).

The balance between potentially violating EFR and allowing an unutilized flow in the system tips toward the latter when shifting from a WF cap set at average BWA to a WF cap set more precautionary at the 25th percentile of BWA. The strictest cap, set at minimum BWA, prevents EFR from being compromised at all times. While included to illustrate potential dynamics of various cap regimes, setting the WF cap so low is arguably unrealistic, especially in basins that already face severe water scarcity. Table 1 quantifies the implications of each of the three procedures to set monthly WF caps for the three river basins discussed earlier. Particularly, the Tigris/Euphrates and Indus basins would experience prolonged periods of consecutive EFR violations even in the middle case in which WF caps are set at the 25th percentile of BWA (4.4 and 3.3 consecutive months on average, respectively). In case of an average BWA‐based cap, in the water‐rich Rhine basin, EFR is potentially violated regularly (6.4 months ever year), but there is always at least a part of EFR remaining (0 months exceed 90% of EFR). This is not the case for the Tigris/Euphrates and Indus basins, where, with an average BWA‐based cap, over 90% of EFR is violated for months at a time (3.8 and 2.7 consecutive months on average, respectively). The monthly WF cap values, as well as the quantified implications in terms of EFR violations and unutilized WF potential as it follows from the three different procedures, are provided in Hogeboom et al. (2019) for all river basins considered.

**Table 1:** Implications of Three Different Procedures to Setting a Monthly WF Caps, for Three River Basins With Distinctive Hydrological Regimes

River basin	WF cap option	Unutilized WF potential		EFR violation^a^				
		10^9^ m^3^ year^−1^	% BWA	10^9^ m^3^ year^−1^	% *BWA*	# mo year^−1^	#c mo	#90 mo
Rhine	avg BWA	6.04	14	6.04	11	6.4	3.6	0
	Q25 BWA	13.2	30	1.52	2.8	2.7	2.1	0
	min BWA	28.3	64	0	0	0	0	0
								
Tigris/	avg BWA	23.3	31	23.3	37	7.6	7.6	3.8
Euphrates	Q25 BWA	46.8	62	2.67	4.2	2.7	4.4	2.0
	min BWA	67.4	90	0	0	0	0	0
								
Indus	avg BWA	25.1	25	25.1	25	7.1	6.9	2.7
	Q25 BWA	51.5	51	4.42	4.4	2.7	3.3	2.3
	min BWA	88.8	87	0	0	0	0	0

*Note*. Long‐term average annual EFR as the percentage of BWR for the Rhine, Tigris/Euphrates, and Indus basins are 56%, 46% and 50%, respectively.

EFR violations are expressed in cubic meter per year; as the percentage of BWA; the average number of months per year (# mo year^−1^) EFR is violated; the average number of consecutive months (#c mo) EFR is violated if a violation occurs; and the average number of months 90% or more of EFR is violated (#90 mo).

## 4 Model Variability and Limitations

**Figure 5:**
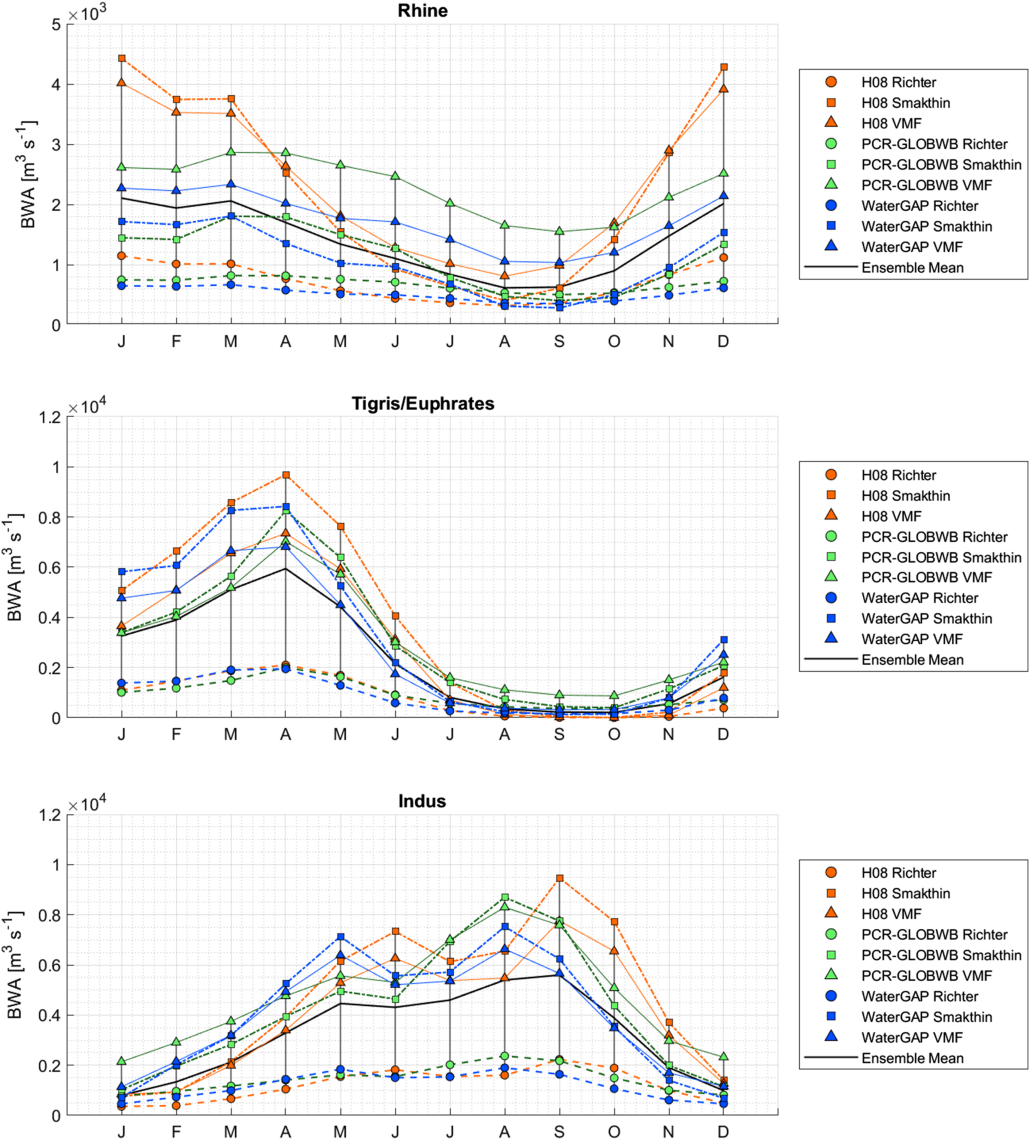
Spread in multiyear BWA for all combinations across three GHMs and three EFR methods, for three selected basins over the period 1970–2005. The black line shows the BWA estimate based on ensemble mean BWR minus ensemble mean EFR.

We set out to quantify monthly blue WF caps for all basins in the world and address the implications of three different procedures to formulate WF caps. First, monthly BWA was estimated per basin by subtracting EFR from BWR, for each month in the period 1970–2005. Ideally, monthly BWA had been based on in situ runoff observations and environmental needs tailored to local basin circumstances rather than modeling. However, until such observations become available with global coverage, modeling can provide a first indication of basin‐level BWA. From the wide spectrum of GHMs and EFR models available, three of each were selected in this study to provide estimates for monthly BWR and EFR. Although taking ensemble means averages out model anomalies or divergencies, Figure 5 reveals a considerable spread across simulated BWA. This spread comes in addition to inherent natural variability in both BWR and EFR.

On the global level, long‐term average BWR based on the three selected GHMs varies between 41,300 and 67,200 km^3^ year^−1^, with an average of 54,100 km^3^ year^−1^. These estimates are comparable to the range of 42,000–66,000 km^3^ year^−1^ found by a previous multimodel runoff assessment by Haddeland et al. (2011)—even though they included anthropogenic water use that, if corrected for, would yield a higher natural runoff estimate. This simulated average of the three selected GHMs is a bit higher than the natural runoff of 49,300 km^3^ year^−1^ reported by Oki and Kanae (2006) and substantially higher than the 41,700 km^3^ year^−1^ given by Gerten et al. (2013). We found that globally aggregated EFR is 26,700 km^3^ year^−1^ on average (with a range of 8,800–53,900 km^3^ year^−1^), or 49% (21%–80%) of BWR. Gerten et al. (2013), who applied five different EFR methods, estimated that globally on average 36% of runoff should be allocated to EFR, with the high end of the range at 57%. The inclusion of the precautionary standard of 80% by Richter et al. (2012) in our selection drives this study's EFR estimate up considerably. Aggregating BWA across basins, we obtain a global total BWA of 27,400 (intermodel range: 8,300–53,700) km^3^ year^−1^ or 187 (intermodel range: 55–366) mm year^−1^.

While intermodel spread could be perceived as a source of uncertainty, each model (and particularly the EFR methods) by itself intends to tell a consistent narrative on runoff routing or EFR. Rather than taking a multimodel mean, it is therefore worth examining a priori which narrative fits the local context best and hence which model or method to choose. Moreover, in presenting our estimates at the basin scale, we failed to include important sub‐basin hydrological dynamics, thereby introducing more uncertainty in these basin's BWA estimates, particularly in the larger drainage basins. In a similar vein, while major flow distortions induced by reservoirs are to some degree included in the GHM routing schemes, we suggest to more precisely investigate reservoir presence, operation, and effect on WF caps. After all, reservoir storage and operations can substantially attenuate basin hydrographs, introduce additional loss terms, and thereby redistribute BWA over time (Di Baldassarre et al., 2018; Hogeboom et al., 2018). A recent study by Zhuo et al. (2019) shows that reservoirs can increase monthly BWA in dry months at the cost of lowering BWA in wet months, occasionally even adding to “scarcity in wet months,” indicating that environmental peak flow requirements were no longer met.

## 5 A Basis for a PB on Water

While our primary intention was to quantify WF caps at the basin level, the estimated monthly BWA figures for the world's river basins—when summed up over the year and all basins—can feed the discourse on PBs (Vanham et al., 2019). Steffen et al. (2015) have proposed 4,000 km^3^ year^−1^ (with an uncertainty range of 4,000–6,000 km^3^ year^−1^) as a PB for global blue freshwater consumption, while acknowledging the need for basin boundaries as well. Gerten et al. (2013) propose a lower PB for water of 2,800 km^3^ year^−1^, with a range of 1,100–4,500 km^3^ year^−1^. Our globally aggregated BWA estimate is much higher. However, since much of BWA runs off during flood periods and/or in areas where not enough people live to use the water, appropriate reductions need to be applied to translate total BWA into an exploitable BWA that is more comparable to these estimates (Postel et al., 1996). Here we do not intend to propose a new PB for freshwater consumption, but rather we illustrate how different rationales will result in a different PB.

For each of the following three rationales, we start with the precautionary principle as proposed by Steffen et al. (2015), by taking for each basin the lower end of the simulated range of BWR and the higher end of the simulated range of EFR (thus yielding the lowest estimate for BWA). This procedure yields in a global BWR of 41,300 km^3^ year^−1^ and a BWA of 8,300 km^3^ year^−1^. The first rationale to assess annual exploitable BWA in every river basin is to equate exploitable BWA in each month to BWA in the most critical month, i.e., the month in which the long‐term average BWA is lowest, and then to sum over the months. Aggregating across basins, this rationale yields a global exploitable BWA of 2,400 km^3^ year^−1^, which can be interpreted as an estimate for the PB for blue water consumption. Alternatively and less strict, we can define exploitable BWA in each month as BWA in that month, but with the baseflow in a river basin as a maximum—here taken as the 5^th^ percentile of monthly BWR over the study period of 1970–2005. Aggregating to the global level, this rationale results in a PB of 3,200 km^3^ year^−1^. As a third assumption, we take the previous rationale but apply an additional remote flow criterion, following Steffen et al. (2015) and Postel et al. (1996). When we subtract all flows in a basin that exceed 1,000 m^3^ cap^−1^ year^−1^, this yields a PB of 1,200 km^3^ year^−1^, viz., the most precautionary value.

The wide range of resulting PB values in response to agile application of assumptions on exploitable flows at the basin level shows that further academic debate is necessary as to which procedure and assumptions to follow in establishing a PB, particularly when using a bottom‐up approach (Gerten et al., 2013). Moreover, the divergence underscores that caution is warranted in interpreting (aggregated) global PB values that have been put forward in literature already. It is clear that regionalized and time‐specific boundaries are more meaningful, in terms of both assessing appropriable volumes and taking action in drafting effective water policies (Heck et al., 2018; Heistermann, 2017; Steffen et al., 2015). At the same time, global PB values and discussions may provide additional, overarching narratives to motivate local policy makers to consider capping WFs within their jurisdiction. Here particularly international actors such as the World Bank and UN agencies can play an agenda‐setting role.

## 6 Toward Policy Uptake

Our work illustrates the intrinsic difficulty of dealing with variability toward any practical policy arrangement, and successful showcases have yet to be developed (Grafton et al., 2014). If WF caps are to become effective and practical concepts for policy arrangements, a number of limitations have to be overcome. Explicitly splitting WF caps into a surface water and renewable groundwater component would be a valuable yet complicating refinement over this study's lumping of the two, as the two sources are often treated differently in water policy. Since the current study represents runoff as the aggregation of both surface water runoff and baseflow, separating WF caps in a surface water and groundwater component would call for an explicit consideration of groundwater extraction potential. Furthermore, and particularly pertaining to larger catchments, the spatial distribution of BWA over the basin complicates arrangements in which a single cap is set for the entire basin as was done in this study. In practice, multiple caps may be needed at sub‐basin scales, ideally aligned with corresponding administrative jurisdictions (Zhou et al., 2019).

The implications of formalizing a WF cap according to different alternative procedures have been expressed here in terms of balancing EFR violations (biodiversity interests) against unutilized WF potential (economic interests). However, there may be additional or other socioecological implications, indicators, or impacts deemed worthy to consider in formulating a capping procedure, e.g., related to ensuring an equitable distribution of water over competing users and communities, or more specific ecological or economic indicators. Which implications and trade‐offs are acceptable will vary across basins. Selecting an appropriate WF capping procedure is therefore to be resolved at the local level, through local policy choices.

It is also in the local setting that potential management, legal, institutional, stakeholder, or governance hurdles can be encountered. For example, adopting a WF cap requires of local (water) authorities that they know—preferably in quantitative terms—local runoff, local environmental water needs, and local water consumption (e.g., through accounting WF permits that are issued to any of the various users at any given time). In ill‐managed or data‐scarce basins, accounting of incoming and outgoing flows will pose a substantial challenge, as does their monitoring (Hogeboom et al., 2015). With respect to institutional hurdles, attempts to incorporate what might be considered a local WF cap in South Africa were stranded in power struggles between various levels of authority (Bourblanc & Blanchon, 2014). Existing legal and governance structures might also obstruct WF capping, for instance, in basins where water rights are tied to land ownership, in basins where WF permits have been issued unconditionally, or in transboundary basins.

Basins with highly variable water availability that already experience severe water stress by overconsumption (e.g., the Indus basin) may have greater challenges in adopting a WF cap regime than moderately stressed basins with less variability (e.g., the Rhine basin). Nevertheless, particularly these highly variable and scarce basins have the strongest imperative to keep a close eye on their water allocation policies. While it is indispensable to curb WFs to cap levels in scarce regions, reduction efforts should, however, not be constrained to severely stressed basins only. After all, a strategic pathway to conserve limited water resources in scarce basins is to increase water productivity in basins that still have the potential for it, e.g., by relocating crops (Pastor et al., 2019; Qin et al., 2019). One means to boost water productivity across basins is to formulate WF benchmarks for water‐using activities, i.e., a reasonable amount of water consumption per activity (Karandish et al., 2018). Another would be to use green water resources more productively (Schyns et al., 2019). Particularly the combination of setting a WF cap per basin, and then issuing WF permits only to those activities that meet their (green and blue) WF benchmark, can be instrumental in achieving truly sustainable and efficient consumption of freshwater worldwide (Hoekstra, 2014).

One avenue for further research that would facilitate practical uptake is to explore whether a dynamic WF cap regime can be developed whereby monthly caps partially depend on runoff forecasts. Authorities can then adapt WF permits to users accordingly, based on near‐real‐time BWA. Ideally, such forecasts capture seasonal‐scale dynamics, as particularly in agriculture decisions on which crops to grow—and thus where WFs will be created—operate on this time scale. Another recommendation is to compare actual WFs to the WF cap values presented here. While in this study we assumed WFs to be equal to the WF cap, taking actual WFs will allow for a more practically relevant assessment of (implications of) setting a WF cap.

## 7 Conclusion

The world's limited blue water resources are shared by humans and nature. The continued growth in human water consumption to produce the products we buy, the food we eat, and the clothes we wear has tremendous impacts on global biodiversity (Vörösmarty et al., 2010). Clearly, bridling humanity's unsustainable WF is one of the key environmental challenges of the 21st century. Capping water consumption, to support the transition toward sustainable freshwater use, is urgent in all river basins where water resources are already overexploited, which concerns about half of the world's basins (Hoekstra et al., 2012).

We have quantified sustainable levels of water consumption by human uses and how they vary over time, for all basins in the world, using several state‐of‐the‐art GHMs and environmental flow methods. While we thus provide a first global assessment, many challenges will need to be overcome to meaningfully embed WF caps in local policy arrangements. We illustrated how setting WF caps calls for seeking a compromise between underutilizing the potential of sustainable water consumption and implicitly accepting violations of EFR—a trade‐off that is particularly pronounced in basins with a high seasonal and interannual variability. Toward practical policy uptake, however, additional indicators to measure implications of using water versus leaving it for nature may be necessary in a given local context. Moreover, authorities will have to agree on which monthly figures of available runoff and environmental flows to consider and which capping procedure to follow—all of which are not straightforward choices.

Despite identified limitations to this study and many hurdles toward implementation at the local basin level that have to be overcome regarding data availability, institutional settings, and existing legal or governance structures, we underscore the evident merit of the concept of capping water consumption in the first place and its valuable contribution to the ongoing quest for sustainable freshwater use worldwide.

## 8 Method

National reports on “annual renewable water resources” as provided by Aquastat (Food and Agriculture Organization, 2016) prove inadequate if we are to capture, at the basin level, intraannual and interannual dynamics of supply. Despite recent attempts to harmonize existing observations (Do et al., 2018), no comprehensive runoff observation systems are in place to provide monthly statistics on maximum sustainable levels of water availability (Syed et al., 2010). For our ambitious global assessment, GHMs are therefore the best means to derive water availability estimates, at the high spatiotemporal resolution required (Gleeson et al., 2012; Shiklomanov, 2000).

### 8.1 Blue Water Availability

Monthly BWA (m^3^ s^−1^) was estimated by subtracting monthly EFR (m^3^ s^−1^) from monthly BWR (m^3^ s^−1^) for all river basins in the world per month in the period 1970–2005.

### 8.2 Blue Water Runoff

BWR was derived from three state‐of‐the‐art GHMs that were included in International Institute for Applied Systems Analysis (IIASA's) Water Futures and Solutions initiative, which aims to establish a consistent set of global water scenarios using similar forcing and input data, thereby facilitating model intercomparison (Wada et al., 2016). The GHMs used here are H08 (Hanasaki et al., 2008), PCR‐GLOBWB (Wada et al., 2014), and WaterGAP (Müller Schmied et al., 2016). Although their routines and algorithms vary, all GHMs operate on a 30 × 30 arcmin spatial resolution with global coverage (except Antarctica); incorporate the GRanD database on major reservoirs (Lehner et al., 2011); are forced with the same historic meteorological time series; and use the flow direction map DDM30 to delineate basins (Döll & Lehner, 2002). We extracted 11,558 unique basins globally, by selecting the end nodes of each basin in DDM30. Many one‐cell coastal basins drive this high number; the bulk of the runoff is accounted for by the ~70 largest basins. Although we are aware that aggregating to the basin scale takes away from the richness available at the grid level, the aim is to illustrate potential dynamics of establishing a WF cap in policy. We therefore reckoned these dynamics to be more clearly demonstrated at the (administrative level of a) drainage basin.

For each basin, monthly natural runoff as computed by the three GHMs—i.e., the natural runoff without water consumption by humans—for each month in the period 1970–2005 was taken to represent BWR. Modeled runoff at these endpoints represents the sum of total (net) streamflow and baseflow generated by the grid cells in the basin, after it is routed to the endnote through the system as it is conceptualized and configured by the respective GHM. Note that the GHMs' basin configurations include man‐made system changes, such as rules for (major) reservoir operations, that affect routing and runoff. Consumptive terms thus generated, e.g., evaporative losses from reservoir surfaces, may be considered anthropogenic. Natural runoff is thus to be understood as runoff in the absense of human consumption but generated by or flowing through human‐altered systems. Differences in model outcomes are known to be a major source of uncertainty (Haddeland et al., 2011); hence, we took the ensemble mean of the three GHMs to obtain a (best‐guess) BWR value. Hogeboom et al. (2019) contains the geometries of 11,493 basins; some basins are not represented in the DDM30 map (Döll & Lehner, 2002) that nevertheless yielded an output for one or more of the GHMs.

### 8.3 Environmental Flow Requirements

An extensive list of environmental flow frameworks has emerged (Brewer et al., 2016), ranging from hydrological and hydraulic frameworks to habitat simulation and more holistic approaches. Required input data at the global level are only readily available for hydrological methods. We therefore selected three such methods to estimate EFR: the Smakhtin method (Smakhtin et al., 2004), the Richter method (Richter et al., 2012), and the variable mean flow (VMF) method (Pastor et al., 2014). The Smakhtin method typically yields low EFR values and distinguishes between high and low flow conditions and allocates a base flow volume represented by the 90^th^ percentile (Q90) of BWR to environmental needs, plus a percentage of the remaining flow depending on the flow regime. Likewise, the VMF method sets apart EFR based on high, intermediate, and low flow regimes, at which between 30% and 60% of BWR is allocated to the environment. The Richter method is the most precautionary method and takes EFR to be a constant percentage of 80% of BWR without distinguishing between flow regimes. Because monthly EFR estimates in both the Smakhtin and VMF methods depend on (annual) basin hydrographs, it can occur that for a particular month EFR exceeds BWR, yielding a negative BWA after EFR is subtracted from BWR. Since this is physically impossible, BWA is set to zero for those particular months. In the Richter method this phenomenon does not occur. Given the known spread between and structural uncertainty of the methods, we took—analogous to the *BWR* estimates—the ensemble mean of the three environmental flow methods to obtain (best‐guess) EFR values.

### 8.4 WF Cap Options and Implications

We drafted three alternative WF cap options, which differ in the procedure followed to formulate WF caps based on the historical BWA statistics. In the first option, a monthly WF cap is set for each basin at the long‐term average of the monthly average BWA over the period 1970–2005. This implies that when actual WFs will equal the level of the caps, WFs will as often exceed the WF cap (thereby violating environmental flows) as underrun it (thereby underutilizing available runoff for human appropriation). In the second option, the monthly WF cap is set at the 25^th^ percentile (Q25) of the monthly average BWA (viz., BWA that is exceeded 75% of the time for a particular month of the year) over the period 1970–2005. In this option, WFs (when equal to the caps) will exceed the cap in fewer occasions than in the previous option, but at the cost of a larger unutilized WF potential. In the third option, the monthly WF cap is set at the minimum monthly BWA that occurred in a particular month during the period 1970–2005. This is the most precautionary definition of what is maximally allowed and implies that total WFs in the basin will always remain below the WF cap. Environmental flows will never be compromised, but the unutilized WF potential will be highest in this option. The implications of the three options are expressed in terms of a trade‐off between potentially allowing environmental flows to be violated versus leaving an unutilized WF potential in the river basin.
